# Highly Variable Chloroplast Markers for Evaluating Plant Phylogeny at Low Taxonomic Levels and for DNA Barcoding

**DOI:** 10.1371/journal.pone.0035071

**Published:** 2012-04-12

**Authors:** Wenpan Dong, Jing Liu, Jing Yu, Ling Wang, Shiliang Zhou

**Affiliations:** 1 State Key Laboratory of Systematic and Evolutionary Botany, Institute of Botany, Chinese Academy of Sciences, Beijing, China; 2 College of Landscape Architecture, Northeast Forestry University, Harbin, China; 3 Graduate University of Chinese Academy of Sciences, Beijing, China; American University in Cairo, Egypt

## Abstract

**Background:**

At present, plant molecular systematics and DNA barcoding techniques rely heavily on the use of chloroplast gene sequences. Because of the relatively low evolutionary rates of chloroplast genes, there are very few choices suitable for molecular studies on angiosperms at low taxonomic levels, and for DNA barcoding of species.

**Methodology/Principal Findings:**

We scanned the entire chloroplast genomes of 12 genera to search for highly variable regions. The sequence data of 9 genera were from GenBank and 3 genera were of our own. We identified nearly 5% of the most variable loci from all variable loci in the chloroplast genomes of each genus, and then selected 23 loci that were present in at least three genera. The 23 loci included 4 coding regions, 2 introns, and 17 intergenic spacers. Of the 23 loci, the most variable (in order from highest variability to lowest) were intergenic regions *ycf1*-a, *trnK*, *rpl32-trnL*, and *trnH-psbA*, followed by *trnS^UGA^-trnG^UCC^*, *petA-psbJ*, *rps16-trnQ*, *ndhC-trnV*, *ycf1*-b, *ndhF*, *rpoB-trnC*, *psbE-petL*, and *rbcL-accD*. Three loci, *trnS^UGA^-trnG^UCC^*, *trnT-psbD*, and *trnW-psaJ*, showed very high nucleotide diversity per site (π values) across three genera. Other loci may have strong potential for resolving phylogenetic and species identification problems at the species level. The loci *accD-psaI*, *rbcL-accD*, *rpl32-trnL*, *rps16-trnQ*, and *ycf1* are absent from some genera. To amplify and sequence the highly variable loci identified in this study, we designed primers from their conserved flanking regions. We tested the applicability of the primers to amplify target sequences in eight species representing basal angiosperms, monocots, eudicots, rosids, and asterids, and confirmed that the primers amplified the desired sequences of these species.

**Significance/Conclusions:**

Chloroplast genome sequences contain regions that are highly variable. Such regions are the first consideration when screening the suitable loci to resolve closely related species or genera in phylogenetic analyses, and for DNA barcoding.

## Introduction

At present, techniques for studying the molecular phylogeny of plants rely heavily on chloroplast genome sequence data. This is because the chloroplast genome has a simple and stable genetic structure, it is haploid, there are no (or very rare) recombination, it is generally uniparentally transmitted, and universal primers can be used to amplify target sequences. Another important reason is the ease of PCR amplification and sequencing of chloroplast genes, despite some intrinsic problems similar to those encountered when using animal mitochondrial DNA [1]. Many fragments of coding regions, introns, and intergenic spacers, including *atpB*, *atpB-rbcL*, *matK*, *ndhF*, *rbcL*, *rpl16*, *rps4-trnS*, *rps16*, *trnH-psbA*, *trnL-F*, *trnS-G*, etc., have been used for phylogenetic reconstructions at various taxonomic levels [2], [3], [4], [5], [6], [7]. Unfortunately, these regions often lack variations in closely related species, especially those that have diverged recently in evolution. Therefore, a concatenation of many individual genes must be used to improve the resolution of the phylogenetic analysis, and to obtain reasonable results. Such extra investments could be avoided if more variable locations were identified and universal primers were available.

Some regions of the chloroplast genome, for example, *atpF-H*, *matK*, *psbK-I*, *rbcL*, *rpoB*, *rpoC1* and *trnH-psbA* have been relied upon heavily for development of candidate markers for plant DNA barcoding [8], [9], [10], [11], [12]. The aim of DNA barcoding is to solve species identification problems, but some regions such as *rbcL*, *rpoB*, and *rpoC1* are useful for identification at the family rather than species level. Recently, candidate loci and some other loci frequently used in phylogenetic analyses were critically evaluated for several flowering plant groups, including *Amomum* [13], *Carex* [14], Meteoriaceae [15], Cycadales [16], *Compsonuera* [17], *Panax* [18], peach [19] and tree peonies [20]. It seems that *matK* and *trnH-psbA* are the two most promising choices of chloroplast regions. The *matK* gene is one of the most versatile candidates so far, because it is useful for identification at family, genus, and even species levels. However, it is difficult to amplify and sequence this region from certain taxa, and additional universal primers and optimization of PCR reactions are necessary [21], [22]. *trnH-psbA* is the most variable region in the chloroplast genome across a wide range of groups. However, there are some exceptions and long mononucleotide repeats (poly-structures or single nucleotide microsatellites) can cause sequencing problems. Another problem is the presence of inversions in the middle of the sequence, which can lead to incorrect alignments [23].

Most of the regions that are commonly used for phylogenetic analyses were first identified in the 1990s, before entire genome sequences were available. Shaw et al. [24] summarized and evaluated the most frequently used chloroplast regions in seed plants, which significantly helped beginning researchers. Currently, about 191 entire chloroplast genomes are available, and some genera have two or more completely sequenced chloroplast genomes. Therefore, it is timely to reevaluate the variability of chloroplast regions at low taxonomic levels. Identification of variable loci in chloroplast genomes will be extremely useful for molecular systematics and DNA barcoding. Many plant species evolved via adaptive radiations or explosive patterns of speciation, and have evolutionary histories of only a few million years. The very short evolutionary histories result in low sequence divergence. The limited sequence variation is usually harbored in a few hotspots, and most of the loci available to researchers based on previous research provide very few informative characters.

To solve phylogenetic problems at the species level, or to identify species using DNA sequences, we need to identify regions with very high evolutionary rates. Greater availability of such regions will increase our ability to resolve such identification problems. Utilization of a larger number of regions of genes or sequences can minimize the noise of the evolutionary heterogeneity of genes or parts of a gene. Therefore, searching for more regions with high evolutionary rates is very important for plant phylogenetic analyses and for DNA barcoding. Fortunately, there are now many complete chloroplast genome sequences available, even for different species in same genera. This information allows the identification of most variable regions between or among species. In this paper, we summarize the results of comparative studies on chloroplast genomes of congeners of flowering plants. Our aim was to find the most variable regions that are common across many genera. Such regions can be used to resolve phylogenies and for DNA barcoding of closely related flowering plant species.

## Results

### Identification of most variable loci in chloroplast genomes

**Figure 1:**
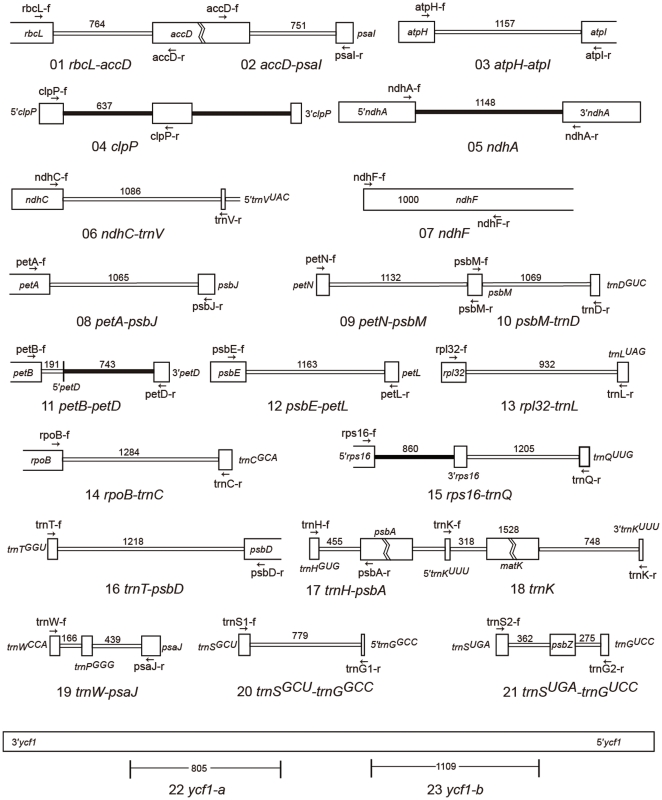
Priming sites of 23 variable regions in chloroplast genome. Large white boxes indicate coding areas, small white boxes indicate intergenic spacers, and small black boxes indicate introns. Figures above boxes indicate length (bp).

There are 14 genera of seed plants in which the chloroplast genome has been sequenced for more than one species. We excluded gymnosperms from analyses because only *Pinus* has several genomes available. Of the angiosperm genera, we excluded *Cuscuta* from our analyses because of drastic genome reorganization and large deletions in the chloroplast genome. We analyzed a total of 12 genera among which nine genera have chloroplast genomic data readily available from GenBank, and the chloroplast genomic data of the other three genera are to be released (Table 1). The maximum number of polymorphic sites (S) within 600 bp in the 12 genera varied from 3 (*Acorus*) to 49 (*Aethionema*) with an arithmetic mean of 22.8. When the regions were restricted by the number of polymorphic sites (S, >
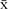
+2stdev), there were 47 highly variable loci present in at least one genus, 29 were shared by two or more genera, 23 by three or more genera, 11 by four or more genera, 10 by 5 or more genera, and only 5 by 6 or more genera. To provide reasonable choices, we further analyzed 23 loci (Table S1). Among them, *ndhF*, *trnK* (containing *matK*), *ycf1*-a, and *ycf1*-b are largely coding regions, *clpP* and *ndhA* are introns, and the other 17 are intergenic spacers (Fig. 1). The most variable locus was *ycf1*, a gene of unknown function. The *ycf1* locus is several kilobase-pairs long. Two regions of *ycf1* showed high variability in 9 of 11 genera, and the π values of the *ycf1* locus in 6 genera were markedly higher than in the other genera. The *rpl32-trnL* and *trnK* (including *matK*) loci were variable in 8 genera, and *rps16-trnQ* and *trnS^UGA^-trnG^UCC^* loci were variable in 6 genera. Judging from the values of nucleotide diversity (π values), *ycf1*, *trnH-psbA*, *rpl32-trnL*, *rps16-trnQ*, and *ndhC-trnV* were the most variable loci with average π values of greater than 0.01 over 12 genera. The other loci showed average π values of greater than 0.0048. The loci *ndhC-trnV*, *rps16-trnQ*, *trnS^UGA^-trnG^GCC^*, *trnS^UGA^-trnG^UCC^*, and *trnT-psbD* were rich in indels. Indels are usually informative in phylogenetic reconstructions and diagnostic to plant taxa.

**Table 1:** Angiosperm genera in which complete chloroplast genomes have been determined in two or more species.

Genus	Species	Family	S_max_	Mean	Stdev
*Acorus*	*A. americanus*	Acoraceae	3	0.82	0.32
	*A. calamus*				
*Aethionema*	*Ae. cordifolium*	Brassicaceae	49	9.67	9.00
	*Ae. grandiflorum*				
*Calycanthus*	*C. chinensis*	Calycanthaceae	10	1.51	1.77
	*C. floridus* var. *glaucus*				
*Chimonanthus*	*Ch. nitens*	Calycanthaceae	10	1.32	1.49
	*Ch. praecox*				
*Eucalyptus*	*E. globulus* subsp. *globulus*	Myrtaceae	10	1.08	1.52
	*E. grandis*				
*Gossypium*	*G. barbadense*	Malvaceae	28	1.44	2.59
	*G. hirsutum*				
*Nicotiana*	*N. sylvestris*	Solanaceae	16	4.00	3.49
	*N. tabacum*				
	*N. tomentosiformis*				
*Oenothera*	*Oe. argillicola*	Onagraceae	42	2.17	3.94
	*Oe. biennis*				
	*Oe. glazioviana*				
	*Oe. parviflora*				
*Oryza*	*O. nivara*	Poaceae	11	0.82	1.43
	*O. sativa* subsp. *indica*				
*Paeonia*	*P. brownii*	Paeoniaceae	31	8.04	5.82
	*P. obovata*				
	*P. suffruticosa*				
*Populus*	*P. alba*	Salicaceae	18	2.02	2.53
	*P. trichocarpa*				
*Solanum*	*S. bulbocastanum*	Solanaceae	26	5.03	4.67
	*S. lycopersicum*				
	*S. tuberosum*				

Maximum number of polymorphic sites (**S_max_**), mean number of polymorphic sites, and standard deviation of polymorphic sites is shown for each genus.

### Universality of primers

**Figure 2:**
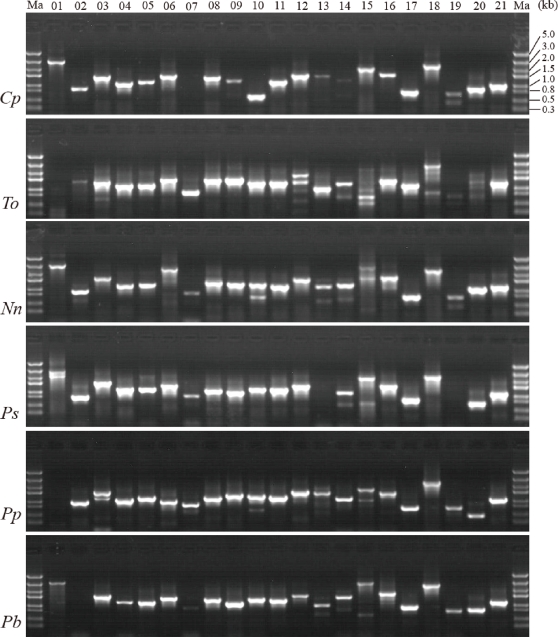
Gel profiles of fragments amplified from six species using 21 pairs of primers. Numbers shown at top are sequential order of loci as in Table 2 and Fig. 1. Numbers on right are size markers (kbp). Letters on left indicate species as follows: *Cp*: *Chimonanthus praecox* (L.) Link; *To*: *Typha orientalis* Presl.; *Nn*: *Nelumbo nucifera* Gaertn.; *Ps*: *Paeonia suffruticosa* Andrews; *Pp*: *Prunus persica* (L.) Batsch.; and *Pb*: *Panax bipinnatifidus* Seem.

Although there are primers available for some loci, e.g., *rps16-trnQ*, *trnH-psbA*, *trnK*, and *trnS^UGA^-trnG^UCC^*, to provide more choices we designed new primers for 21 loci (Fig. 1, Table 2) based on available chloroplast genome data from GenBank. The two regions of *ycf1* were too long and variable to design universal primers. All new primers were tested using eight species covering basal angiosperms, monocots, eudicots, rosids, and asterids. The primer pairs for 15 loci showed complete amplification successes with the eight testing species (Table 2). For some other primer pair/species combinations we failed to amplify sequences as follows: *rbcL-accD* of *Typha orientalis* and *Prunus persica*, *accD-psaI* of *Panax bipinnatifidus*, *ndhF* of *Chimonanthus praecox*, *rpl32-trnL* of *Paeonia suffruticosa*, *trnS^GCU^-trnG^GCC^* of *T. orientalis*, and *trnW-psaJ* of *P. suffruticosa* (Fig. 2). To check the quality of amplified fragments, PCR products from *Nelumbo nucifera*, *Prunus mira*, and *Panax bipinnatifidus* were purified with PEG8000 and directly sequenced. We considered 600 bp to be an acceptable length of a read for the sequence of a given product. One hundred and ninety-eight reads of the 210 in total (∼94.3%) reached 600 bp in length and had quality values (ratios of bases with QV >20 to the total bases of a read) higher than 90% after trimming both ends (Table 2).

**Table 2:** Primers for amplifying and/or sequencing 23 highly variable loci.

	Locus	AS	Forward primer				Reverse primer			
			Name	Sequence 5′to 3′	SS	Q	Name	Sequence 5′to 3′	SS	Q
1	*rbcL-accD*	66.67	rbcL-f	tagctgctgcttgtgaggtatgga	100	96.1–98.0	accD-r	aaatactaggcccactaaagg	100	96.7–97.0
2	*accD-psaI*	83.33	accD-f	ggtaaaagagtaattgaacaaac	100	90.9–99.5	psaI-r	ggaaatactaagcccactaaaggcaca	100	99.1–99.4
3	*atpH-atpI*	100	atpH-f	aacaaaaggattcgcaaataaaag	100	98.1–99.5	atpI-r	agttgttgttcttgtttctttagt	85.71	97.5–99.2
4	*clpP*	100	clpP-f	gcttgggcttctcttgctgacat	71.43	98.2–98.8	clpP-r	tcctaatcaaccgactttatcgag	85.71	95.7–98.8
5	*ndhA*	100	ndhA-f	tcaactatatcaactgtacttgaac	100	97.8–99.1	ndhA-r	cgagctgctgctcaatcgat	100	97.3–99.2
6	*ndhC-trnV*	100	ndhC-f	agaccattccaatgccccctttcgcc	100	97.8–99.1	trnV-r	gttcgagtccgtatagcccta	100	97.5–98.4
7	*ndhF*	83.33	ndhF-f	acaccaacgccattcgtaatgccatc	100	98.3–99.1	ndhF-r	aagatgaaattcttaatgatagttgg	100	98.7–99.5
8	*petA-psbJ*	100	petA-f	ggatttggtcagggagatgc	100	97.3–99.2	psbJ-r	atggccgatactactggaagg	85.71	93.5–98.9
9	*petN-psbM*	100	petN-f	atggatatagtaagtctcgcttgg	100	96.5–98.3	psbM-r	atggaagtaaatattcttgcat	100	95.0–98.7
10	*psbM-trnD*	100	psbM-f	tttgactgactgtttttacgta	100	97.6–99.2	trnD-r	cagagcaccgccctgtcaag	100	97.5–99.6
11	*petB-petD*	100	petB-f	caatccactttgactcgtttt	100	97.8–98.9	petD-r	ggttcaccaatcattgatggttc	100	97.7–98.8
12	*psbE-petL*	100	psbE-f	atctactaaattcatcgagttgttcc	100	93.2–98.9	petL-r	tatcttgctcagaccaataaataga	100	94.4–98.8
13	*rpl32-trnL*	83.33	rpl32-f	gcgtattcgtaaaaatatttggaa	100	97.2–99.3	trnL-r	ttcctaagagcagcgtgtctacc	80	96.0–98.6
14	*rpoB-trnC*	100	rpoB-f	acaaaatccttcaaattgtatctga	75	96.9–99.0	trnC-r	tttgttaatcaggcgacacccgg	100	91.7–98.9
15	*rps16-trnQ*	100	rps16-f	tttatcggatcataaaaacccact	80	96.0–98.7	trnQ-r	tggggcgtggccaagcggt	80	95.3–99.1
16	*trnT-psbD*	100	trnT-f	gcccttttaactcagtggtagag	71.43	93.9–99.1	psbD-r	ccaaataggaactggccaatc	100	98.6–99.1
17	*trnH-psbA*	100	trnH-f	cgcgcatggtggattcacaaatc	100	97.7–99.1	psbA-r	tgcatggttccttggtaacttc	100	98.5–99.4
18	*trnK*	100	trnK-f	gggactcgaacccggaacta	100	98.0–99.1	trnK-r	agtactcggcttttaagtgcg	100	88.7–98.8
19	*trnW-psaJ*	83.33	trnW-f	tctaccgaactgaactaagagcgc	100	98.5–99.1	psaJ-r	cgattaatctctatcaatagacctgc	100	96.3–99.1
20	*trnS^GCU^-trnG^GCC^*	83.33	trnS1-f	aacggattagcaatccgacgcttta	100	98.0–98.8	trnG1-r	cttttaccactaaactatacccgc	100	97.5–98.8
21	*trnS^UGA^-trnG^UCC^*	100	trnS2-f	cggttttcaagaccggagctatcaa	100	94.6–98.9	trnG2-r	cataaccttgaggtcacgggttcaaat	71.43	98.1–98.9

AS: PCR amplification success (%); SS: sequencing success (%); Q: percentage of bases with QV (quality value) >20.

### Variability of 21 loci across *Nelumbo*, *Panax*, and *Prunus*, and comparisons with other loci

**Figure 3:**
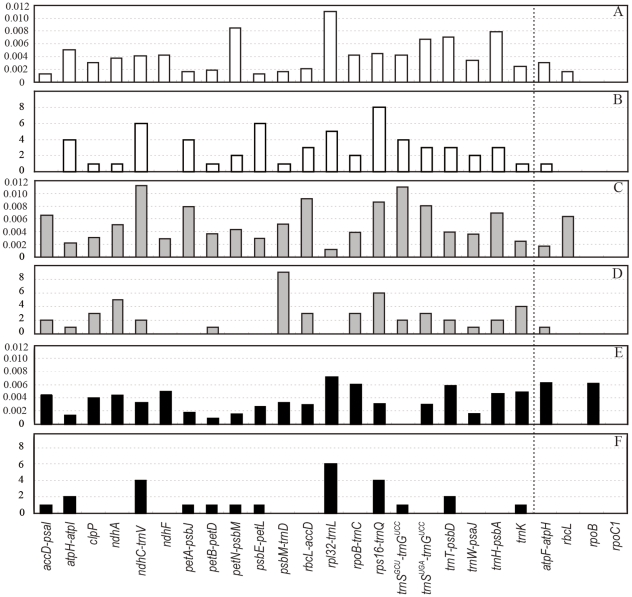
Nucleotide diversity per site (*π*) and indels and inversions (*I*) of 21 loci in *Nelumbo*, *Panax*, and *Prunus*. A & B, *Nelumbo*, C & D *Panax*, E & F, *Prunus*. A, C & E, *π*; B, D & F I. Four proposed barcoding loci, *atpF-atpH*, *rbcL*, *rpoB*, and *rpoC1* were used as controls.

To assess the variability of the 21 loci, we selected two genera, *Panax* and *Prunus*, which have been studied for DNA barcoding purposes [18], [19], and the family Nelumbonaceae, which contains only two species. The number of variable sites, the nucleotide diversity, and the number of indels plus inverses were used as indicators. Four loci that have been suggested as candidate barcodes (*atpF-H*, *rbcL*, *ropB*, and *rpoC1*) were used as controls. The 21 loci performed satisfactory although not all of them were better than the controls in all three genera (Fig. 3).

### Performances of the 21 loci, a case study on peaches

**Figure 4:**
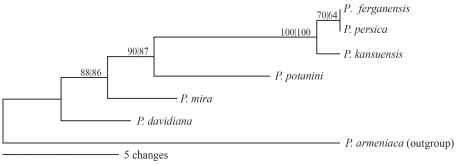
Maximum parsimony tree of *Prunus* sect. *Persica* based on a concatenation of *psbM-trnD* intergenic spacer and *clpP* intron, two representatives of the 21 loci. The figures above the branches are the bootstrap values (NJ|MP) of the clades.

Peaches are a natural group of five or six species belonging to *Prunus* L. sect. *Persica* (L.) S. L. Zhou & X. Quan [19] or *Amygdalus* subg. *Persica* [25]. Nine chloroplast loci had been evaluated for DNA barcoding purpose [19]. The bootstrap consensus trees constructed using maximum parsimony based on the 21 loci (Fig. S1) show moderate to high resolutions. A combination of *psbM-trnD* intergenic spacer and *clpP* intron can solve all six species (Fig. 4).

## Discussion

It is believed that there are mutationally (or substitutionally) active regions in genomes, and our genomic survey of 12 genera indicated that such regions exist in chloroplast genomes. The number of polymorphic sites (S values) varied from 3 to 49 (Table 1) with an arithmetic mean value of 22.8 in 600 bp, indicating great potential for finding variable regions carrying phylogenetic information. Some considerably higher sequence variability of introns and intergenic spacers in chloroplast genomes has been reported (e.g. [24], [26], [27]). Mutationally active regions in chloroplast genomes are frequently regarded as problematic for phylogenetic analyses at higher taxonomic levels because of recombination and sequence convergence, although there are conflicting opinions on the issue (e.g. [28]). However, at lower taxonomic levels of flowering plants, the problems are less serious because in most cases there is insufficient sequence variation in chloroplast genes, rather than the high homoplasy that results from supervariability, as is found in mitochondrial genes.

The most variable locus was *ycf1*, a gene of unknown function. It is more variable than the *matK* locus in the Orchidaceae [29]. The *ycf1* locus is several kb long. The region locates in the IRb region is conservative and two regions located in SSC region are extremely variable, and thus suitable for phylogenetic studies or DNA barcoding at low taxonomic levels. Unfortunately, because of the vast sequence differences at the *ycf1* locus, we could not obtain universal primers currently. It is worthy of working out universal primers after examining more sequences or taxon-specific primers such as *matK* [30]. The *matK* region alone or together with *trnK* introns has been extensively used in molecular systematics and suggested to be a barcode for plants [6], [31]. However, the variability of *matK* region is not as remarkable as some other loci in the genera we examined, except for in *Chimonanthus*. Therefore, the *matK* locus could be helpful in separating angiosperm families or genera but very rarely species. The 3′ region of *ndhF* locus is more variable than the 5′ region and such a kind of variations is suitable for phylogenetic reconstruction for either old or recent groups [7]. The 3′ region of *ndhF* exhibited relatively high variability in *Chimonanthus*, *Eucalyptus*, *Populus* and *Prunus* (Table S1, Fig. 3E). The locus *rpl32-trnL* showed considerable length variation across taxa and a high level of positional variability. If the sequences can be unambiguously aligned, the *rpl32-trnL* region will be suitable for species identification. Unfortunately, the *rpl32-trnL* region has rarely been examined in systematics, and more case studies would further clarify its suitability for such analyses. The *trnH-psbA* was suggested as a candidate DNA barcode early [8], but has not been bolstered in subsequent studies. The *trnH-psbA* locus is really variable in most cases but suffers short length and, therefore, may not provide enough informative characters. Moreover, inversions or mononucleotide repeats are likely to exist at the *trnH-psbA* locus, which may result incorrect alignments or bring sequencing difficulties. The *trnS-trnG* has been well analyzed by Shaw et al. [24] and primer sequences were provided. The *trnS* ^UGA^-*trnG* ^UCC^ includes *psbZ*. So the *trnS* ^GCU^-*trnG* ^GCC^ region is likely to be more variable than *trnS* ^UGA^-*trnG* ^UCC^. Unexpectedly the variability of the two regions is taxon-dependent. The major problems are polyT sequences in the *trnS* ^GCU^-*trnG* ^GCC^ region and (AT)n elements in the *trnS* ^UGA^-*trnG* ^UCC^ region. A large indel in the *trnS* ^UGA^-*trnG* ^UCC^ region was found variable within species [19]. Although the *trnS* ^UGA^ was considered to be of mitochondrial origin for majority of taxa [32], it is also present in chloroplast genomes and the intergenic spacer *trnS* ^UGA^-*trnG* ^UCC^ should be unique to the chloroplast genomes. The *petA-psbJ* and *rps16-trnQ* was variable in such genera as *Acorus*, *Paeonia* and *Oryza* in which very few variable regions exist. Similar to *trnH-psbA* indels and inversions are likely to happen in *petA-psbJ*. The loci *ndhC-trnV*, *ndhF*, *rpoB-trnC*, *psbE-petL* and *rbcL-accD* have been tried occasionally with varying successes. Among them the *rbcL-accD* deserves more attention because the *rbcL* has been suggested to be a barcode together with *matK* [31]. The discrimination power of *rbcL* is not as high as *matK*. Inclusion of *rbcL-accD* would compensate the insufficient variations of *rbcL*. The 10 other loci shown in Table S1 were variable in 3 genera. Some showed high π values in some genera, indicating that they could be useful for resolving phylogenetic relationships in those taxa.

Most of the loci identified in this study have been used frequently for phylogenetic reconstructions, and their evolutionary features have been discussed [24], [26], [27]. Some of the loci, e.g., *clpP*, *petB-petD* [33], *rbcL-accD* [33], [34], and *trnW-psaJ* have only been used occasionally. The two introns of *clpP* have seldom been considered in phylogenetic studies. The first intron of *clpP* exhibits moderate variability in some taxa, and has some potential applications, however, it may be absent from some taxa [35], [36]. The *trnW-psaJ* locus includes two intergenic spacers and a coding region (*trnW-trnP-psaJ*). The *trnW-psaJ* region has never been used independently and its variability should be evaluated. The *trnW-psaJ* region was the most variable region in *Acorus*, and was also one of the most variable regions in *Eucalyptus* (Table S1). A complete assessment of the loci presented in this study should be conducted before making final decisions about markers used for analyses.

In rapidly evolving regions of the chloroplast genome, evolutionary events that occur include the formation of secondary structures, multiple-hit sites, and intra-molecular recombination events. These problems seem less serious in phylogenetic analyses of closely related species. However, to be frank our aim to accurately solve phylogenetic relationships by using the loci identified in this study may not always be achieved because of other problems. For example, the loci *accD-psaI*, *rbcL-accD*, *rpl32-trnL*, *rps16-trnQ*, and *ycf1* are likely to be absent from some genera, which limits their applications. Minute inversions are often observed in rapidly evolving regions such as introns and intergenic spacers, e.g., *trnH-psbA*, *petN-psbM* [23], [37]. If such structures were not recognized, the resulting alignments could be problematic. In addition, incorrect use of information regarding length variation could lead to misguided conclusions, because it can be very difficult to determine homologies among sequences that vary substantially in length. Both mononucleotide and multinucleotide repeats (microsatellites, such as in *psbM-trnD*, *trnS^GCU^-trnG^GCC^* and *trnS^UGA^-trnG^UCC^*) are sometimes present in rapidly evolving regions, and the exact numbers of repeats are difficult to determine by direct sequencing of PCR products. Several authors have suggested ways to interpret the informative but problematic characters [38], [39].

The variability of chloroplast genes differs markedly among genera (Fig. 3). There are intrinsic difficulties and/or taxonomic problems in finding variable regions in chloroplast genomes. The sequence divergence between the two species in *Acorus* is so small that the maximum number of substitutions every 600 bp was only 3, compared with 49 in *Aethionema*. *Acorus americanus* and *A. calamus* are well diverged morphologically. The very small sequence difference between *A. americanus* and *A. calamus* is perhaps due to short time of divergence. *Oryza sativa* subsp. *indica* and *O. nivara* are also very closely related. The cultivated *O. sativa* originated from *O. rufipogon* and/or *O. nivara* a few thousand years ago as a result of human selection [40]. Therefore, the variation between *O. sativa* subsp. *indica* and *O. nivara* is more properly considered intraspecific variation and it is unlikely that regions with high π values will be found. For such taxa, a combination of multiple genes is necessary to uncover more informative characters. In our case of peaches, many loci resolve the species better than *matK* (*trnK*), *rbcL* or *trnH-psbA* (Fig. S1) and a combination of *psbM-trnD* and *clpP* intron can resolve all six species (Fig. 4).

Although candidate genes have been significantly narrowed by this study, the loci we suggest may not be applicable for all flowering plants; however, most of the identified loci are good initial candidates for further evaluations. The controversies regarding plant DNA barcodes will continue even after markers become mandatory. Taxon-specific markers will have to be used for some difficult-to-differentiate taxa. In addition, markers that worked well in one case study do not guarantee suitability for another. Thus, pilot studies are necessary for any untested taxa and further assessments are required to better determine which loci will be useful for any given taxonomic and/or DNA barcoding questions. Moreover, if none of the candidates listed in Table S1 proves satisfactory, we have shown that additional choices are available for potential exploration (Table S3).

## Methods

### Collection of congener genome data and identification of variable loci

We downloaded from GenBank all chloroplast genome sequences in genera with at least two different species (Table 1). In our analyses, we also included six newly determined chloroplast genome sequences, three from the Calycanthaceae and three from Paeoniaceae (to be published). The sequences were first aligned using ClustalX 2.0 [41], and then manually adjusted with Se-Al 2.0a11 [42]. Inversions, if present, were separated to avoid exaggerated sequence differences. The variability of the aligned genomes was evaluated using the sliding window method in DNAsp ver. 4.5 [43]. The window length was set to 600 base pairs (bp), the typical length of DNA barcodes. The step size was set to 50 for relatively accurate positioning of variable regions. We only considered regions with the number of polymorphic sites (S) >
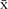
+2 stdev. The regions were identified according to the original annotations, then extracted and compared among the genera after precise alignments. Regions were excluded from further consideration if they were present in fewer than three genera.

### Primer design, applicability tests, and variability assessment

The locations of highly variable regions were precisely identified, and the conserved sequences flanking the regions were used for primer design. Primers for amplifying highly variable regions were designed using Primer Premier v. 5.0 (Premier Biosoft International, CA, USA) and Oligo v. 6.71 (Molecular Biology Insights, CO, USA). The primer pairs were synthesized by Sangon Biotech (Shanghai) Co. Ltd. (Beijing, China).

We used eight species representing basal angiosperms, monocots, eudicots, rosids, and asterids (Table S2) to test the applicability of the primers and the variability of the selected loci. Total DNA was extracted by the CTAB method [44] from silicon gel-dried materials (Table S2). Polymerase chain reactions (PCR) were carried out in 20 µL reaction mixtures. Each PCR mixture contained 2.0 µL 10×buffer, 2.0 µL dNTPs (2 µmol/L), 1.0 µL each primer (5 µmol/L), 1.0 µL total DNA (∼25 ng), 0.2 µL Taq polymerase (5 U/µL), and 11.8 µL ddH_2_O. The PCR program was as follows: 94°C for 3 min, followed by 34 cycles of 94°C for 30 s, 52°C (regardless of the Tm values) for 30 s, 72°C for 2 min, with final extension at 72°C for 5 min. PCR amplifications were carried out using a Veriti 96 Well Thermal Cycler (Applied Biosystems, Foster City, CA, USA). Six species from three genera presenting basal eudicots, rosids and asterids were used to evaluate the variability of the loci and all of the resulting fragments were sequenced on an Applied Biosystems 3730*xl* DNA Analyzer (Applied Biosystems, Foster City, CA, USA), following the manufacturer's instructions. The sequences were assembled using Sequencer 4.7 (Gene Codes, Ann Arbor, MI, USA), aligned with ClustalX [45], and adjusted manually with Se-Al 2.0 [42]. The number of polymorphic sites and nucleotide diversity per site (π) were computed using DnaSP ver. 5.10 [43].

### Case study

Considering very low sequence variations within species [19], sequences of all 21 loci from all six species were used to test the resolutions of the loci. The species [vouchers in parenthesis, please refer to Quan and Zhou [19] for more detailed information] are *P. davidiana* (QX095), *P. ferganensis* (QX020), *P. kansuensis* (QX026), *P. mira* (QX138), *P. persica* (QX048), and *P. potanini* (SL4805–83), and *P. armeniaca* (SL4802–69) was used as an outgroup. *Prunus ferganensis* considered to be a distinct species instead of treating it a subspecies in *P. persica* as Quan and Zhou [19]. The methods for obtaining and analyzing the sequences are the same as above. The resolution of each locus was judged by the maximum parsimonious trees built with PAUP* [46] with the same settings as Quan and Zhou [19].

## Supporting Information

Table S1 The twenty-three most variable regions in chloroplast genomes of 12 genera with two or more species.
(DOC)

Table S2 Samples used to test the of 23 chloroplast loci.
(DOC)

Table S3 Twenty-four loci of high potentials. The values are nucleotide diversity per site (π).
(XLS)

Figure S1 Maximum parsimony trees of all six peach species (*Prunus* sect. *Persica*) based on 21 chloroplast loci, showing the resolutions of the loci in the group. The figures above the lines are the bootstrap values for the clades.
(PDF)
