# Supplementary material for: Highly Variable Chloroplast Markers for Evaluating Plant Phylogeny at Low Taxonomic Levels and for DNA Barcoding
Source: PLoS One. 2012 Apr 12;7(4):e35071. doi: 10.1371/journal.pone.0035071 (PMC3325284; doi:10.1371/journal.pone.0035071)
Supplement: Table S1 — The twenty-three most variable regions in chloroplast genomes of 12 genera with two or more species. (DOC) [file pone.0035071.s001.doc]

**Table S1**. The twenty-three most variable regions in chloroplast genomes of 12 genera with two or more species.

| Genus |  | *Acorus* | *Aethionema* | *Calycanthus* | *Chimonanthus* | *Eucalyptus* | *Gossypium* | *Nicotiana* | *Oenothera* | *Oryza* | *Paeonia* | *Populus* | *Solanum* |
| --- | --- | --- | --- | --- | --- | --- | --- | --- | --- | --- | --- | --- | --- |
| *accD-psaI* | L | - | 849-850 | 829-845 | 834 | 939-942 | 952 | 950 | - | - | 733 | 891-916 | 943-945 |
|  | π | - | 0.0191 | 0.0084 | 0.0036 | 0.0043 | 0.0032 | 0.0084 | - | - | 0.0277 | 0.0034 | 0.0089 |
|  | I | - | 3 | 0 | 0 | 0 | 0 | 0 | - | - | 0 | 4 | 1 |
| *atpH-atpI* | L | 927 | 494-503 | 991-1081 | 1077 | 1108-1110 | 1255-1265 | 1156-1164 | 933-938 | 793 | 1173-1176 | 1008-1025 | 1156-1162 |
|  | π | 0 | 0.0304 | 0.0061 | 0.0028 | 0.0036 | 0.0016 | 0.0029 | 0.0047 | 0 | 0.0154 | 0.0060 | 0.0082 |
|  | I | 0 | 2 | 1 | 0 | 0 | 2 | 2 | 1 | 0 | 1 | 1 | 3 |
| *clpP* | L | 886 | 836-837 | 863 | 865-870 | 837-865 | 923-927 | 878-881 | 250 | 243 | 898-901 | 872-874 | 861-869 |
|  | π | 0 | 0.0180 | 0 | 0 | 0.0036 | 0.0011 | 0.0023 | 0.0040 | 0 | 0.0126 | 0.0034 | 0.0109 |
|  | I | 0 | 0 | 0 | 0 | 1 | 1 | 0 | 0 | 0 | 0 | 0 | 2 |
| *ndhA* | L | 1172 | 1154-1160 | 1166 | 1175 | 1127-1133 | 1143 | 1215 | 1111-1134 | 1055 | 1078-1084 | 1138-1192 | 1200-1225 |
|  | π | 0 | 0 | 0.0043 | 0.0034 | 0.0044 | 0.0018 | 0.0077 | 0.0023 | 0.0010 | 0.0180 | 0.0044 | 0.0106 |
|  | I | 0 | 0 | 0 | 0 | 0 | 0 | 0 | 1 | 0 | 0 | 1 | 2 |
| *ndhC-trnV* | L | 1892 | 841-847 | 1152-1157 | 1157 | 494 | 854-860 | 1019-1066 | 897 | 687 | 966-987 | 1282-1284 | 841-1076 |
|  | π | 0 | 0.0408 | 0.0035 | 0.0035 | 0.0041 | 0.0059 | 0.0119 | 0.0019 | 0.0073 | 0.0237 | 0.0109 | 0.0122 |
|  | I | 0 | 2 | 1 | 0 | 0 | 1 | 3 | 0 | 0 | 4 | 12 | 3 |
| *ndhF* | L | 965 | 971 | 974 | 974 | 985 | 953 | 962 | 980 | 959 | 962-968 | 1007 | 962 |
|  | π | 0 | 0.0309 | 0.0062 | 0.0072 | 0.0071 | 0.0011 | 0.0090 | 0 | 0 | 0.0208 | 0.0079 | 0.0097 |
|  | I | 0 | 0 | 0 | 0 | 0 | 0 | 0 | 0 | 0 | 1 | 0 | 0 |
| *petA-psbJ* | L | 850 | 936-937 | 1170-1173 | 1167-1174 | 967 | 1085-1089 | 1017-1037 | 920-1049 | 977 | 810-835 | 917-925 | 1040-1063 |
|  | π | 0.0012 | 0.0307 | 0.0068 | 0.0026 | 0.0021 | 0.0009 | 0.0098 | 0.0013 | 0 | 0.0374 | 0.0055 | 0.0066 |
|  | I | 0 | 6 | 0 | 1 | 1 | 3 | 1 | 3 | 0 | 7 | 0 | 5 |
| *petB-petD* | L | 1034 | 1035 | 1015 | 1015 | 1080 | 1080-1083 | 1050 | 1070-1083 | 1057 | 1021-1024 | 1139-1142 | 1050-1059 |
|  | π | 0 | 0.0213 | 0.0049 | 0.0049 | 0.0009 | 0 | 0.0102 | 0.0014 | 0.0038 | 0.0151 | 0.0009 | 0.0057 |
|  | I | 0 | 2 | 0 | 0 | 0 | 0 | 0 | 1 | 0 | 2 | 0 | 1 |
| *petN-psbM* | L | 1017 | 548-563 | 1098 | 1095 | 996-999 | 1164-1215 | 1169-1171 | 963 | 797 | 873-884 | 1315-1349 | 1144-1150 |
|  | π | 0 | 0.0368 | 0.0064 | 0.0037 | 0.0040 | 0.0009 | 0.0069 | 0.0023 | 0 | 0.0139 | 0.0038 | 0.0100 |
|  | I | 0 | 2 | 0 | 0 | 0 | 1 | 3 | 0 | 0 | 3 | 4 | 2 |
| *psbE--petL* | L | 1186 | 1105-1265 | 1144-1152 | 1188-1191 | 1178-1198 | 768-770 | 1035-1079 | 890-1306 | 1106 | 1019-1051 | 1080-1093 | 904-913 |
|  | π | 0.0008 | 0.0384 | 0.0052 | 0.0042 | 0.0026 | 0.0070 | 0.0077 | 0.0019 | 0 | 0.0278 | 0.0047 | 0.00221 |
|  | I | 0 | 2 | 1 | 3 | 1 | 1 | 2 | 0 | 0 | 4 | 3 | 1 |
| *psbM-trnD* | L | 528 | 967-982 | 483 | 483 | 1106-1110 | 516 | 1074-1085 | 364-369 | 386 | 449-457 | 1216-1238 | 365 |
|  | π | 0 | 0.0445 | 0.0041 | 0.0062 | 0.0018 | 0 | 0.0037 | 0.0028 | 0 | 0.0120 | 0.0107 | 0.0037 |
|  | I | 0 | 8 | 0 | 0 | 0 | 0 | 1 | 1 | 0 | 1 | 3 | 0 |
| *rbcL-accD* | L | 820 | 748-768 | 734-739 | 748 | 770 | 786-802 | 877-880 | - | - | 803-812 | 699-712 | 856-863 |
|  | π | 0 | 0.0322 | 0.0014 | 0.0054 | 0.0039 | 0.0090 | 0.0084 | - | - | 0.0227 | 0.0043 | 0.0048 |
|  | I | 0 | 3 | 1 | 0 | 0 | 2 | 4 | - | - | 4 | 1 | 3 |
| *rpl32-trnL* | L | 893 | 523 | 1393 | 1400 | 612-620 | 1152 | 997 | 363-382 | 445 | 257-346 | - | 649-953 |
|  | π | 0 | 0.0561 | 0.0079 | 0.0079 | 0.0033 | 0.0017 | 0.0137 | 0.0061 | 0 | 0.0130 | - | 0.0248 |
|  | I | 0 | 2 | 1 | 1 | 0 | 0 | 1 | 2 | 0 | 1 | - | 4 |
| *rpoB-trnC* | L | 1079 | 1135-1139 | 1174-1183 | 1178-1186 | 1284-1297 | 1238-1242 | 1321-1330 | 1250-1263 | 1120 | 991-994 | 1176-1255 | 1350-1349 |
|  | π | 0 | 0.0265 | 0.0026 | 0.0017 | 0.0008 | 0.0032 | 0.0081 | 0.0015 | 0 | 0.0175 | 0.0086 | 0.0089 |
|  | I | 0 | 4 | 1 | 2 | 2 | 1 | 1 | 1 | 0 | 1 | 5 | 0 |
| *rps16-trnQ* | L | 1757 | 1456-1474 | 2081-2087 | 2068-1083 | 2361 | 1638 | 2106-2174 | - | 1980-1986 | 2209-2237 | - | 2030-2053 |
|  | π | 0 | 0.0463 | 0.0053 | 0.0053 | 0.0021 | 0.0055 | 0.0106 | - | 0.0015 | 0.0190 | - | 0.0103 |
|  | I | 0 | 5 | 0 | 1 | 0 | 2 | 5 | - | 1 | 4 | - | 5 |
| *trnH-psbA* | L | 514 | 338-399 | 413 | 413 | 588-634 | 480-487 | 553-581 | 397-408 | 665 | 434-448 | 365-371 | 565-585 |
|  | π | 0 | 0.0481 | 0.0097 | 0.0048 | 0.0051 | 0.0042 | 0.0169 | 0.0013 | 0 | 0.0316 | 0.0110 | 0.0201 |
|  | I | 0 | 3 | 0 | 0 | 2 | 1 | 2 | 1 | 0 | 3 | 0 | 1 |
| *trnK* | L | 2433 | 2499-2500 | 2405 | 2405 | 2845-2488 | 2468-2475 | 2459 | 2404-2409 | 2420 | 2381-2385 | 2488-2490 | 2434-2447 |
|  | π | 0 | 0.0361 | 0.0058 | 0.0058 | 0.0040 | 0.0024 | 0.0082 | 0.0022 | 0 | 0.0125 | 0.0032 | 0.0077 |
|  | I | 0 | 2 | 0 | 0 | 0 | 1 | 2 | 0 | 0 | 2 | 2 | 1 |
| *trnS^GCU^-trnG^GCC^* | L | 1624 | 1195-1198 | 1537 | 1542-1546 | 1458-1496 | 1595-1597 | 1411-1415 | 1524-1594 | 771 | 1278-1306 | 1372-1385 | 1259-1274 |
|  | π | 0 | 0.0322 | 0.0026 | 0.0065 | 0.0021 | 0.0057 | 0.0090 | 0.0021 | 0 | 0.0149 | 0.0133 | 0.0064 |
|  | I | 0 | 5 | 0 | 1 | 3 | 0 | 3 | 7 | 0 | 8 | 3 | 4 |
| *trnS^UGA^-trnG^GCC^* | L | 1058 | 1249-1254 | 951 | 949 | 1474-1486 | 1499-1614 | 1122-1126 | 1128-1191 | 1180-1185 | 1026-1060 | 1409-1426 | 1099 |
|  | π | 0 | 0.0187 | 0.0095 | 0.0074 | 0.0048 | 0.0047 | 0.0024 | 0.0014 | 0.0009 | 0.0124 | 0.0067 | 0.0055 |
|  | I | 0 | 3 | 0 | 0 | 1 | 6 | 2 | 2 | 0 | 4 | 11 | 0 |
| *trnT-psbD* | L | 1498 | 1284-1328 | 1513-1571 | 1510 | 1549-1580 | 1570-1584 | 1327-1349 | 1527-1559 | 756 | 1379-1385 | 1570 | 1304-1310 |
|  | π | 0.0013 | 0.0248 | 0.0054 | 0.0033 | 0.0052 | 0.0013 | 0.0080 | 0.0018 | 0 | 0.0121 | 0.0039 | 0.0031 |
|  | I | 0 | 5 | 3 | 0 | 3 | 2 | 2 | 4 | 0 | 1 | 3 | 1 |
| *trnW-psaJ* | L | 648 | 692-694 | 632 | 632 | 738 | 667-669 | 716-728 | 768-826 | 571 | 583-629 | 770 | 721 |
|  | π | 0.0013 | 0.0264 | 0.0054 | 0.0033 | 0.0052 | 0.0013 | 0.0080 | 0.0018 | 0 | 0.0121 | 0.0039 | 0.0031 |
|  | I | 0 | 1 | 0 | 0 | 0 | 1 | 1 | 4 | 0 | 2 | 0 | 1 |
| *ycf1-a* | L | 831 | 770-776 | 804 | 792 | 819 | 885 | 799-817 | 932-941 | - | 781 | 841 | 829-835 |
|  | π | 0 | 0.0636 | 0.0112 | 0.0025 | 0.0024 | 0.0023 | 0.0134 | 0.0018 | - | 0.0256 | 0.0095 | 0.0161 |
|  | I | 0 | 1 | 0 | 0 | 0 | 0 | 1 | 1 | - | 0 | 0 | 1 |
| *ycf1-b* | L | 1139 | 1046-1049 | 1100 | 1094 | 1043 | 1043-1049 | 1109 | 1352-1445 | - | 1048-1069 | 1090-1123 | 1073-1079 |
|  | π | 0 | 0.0583 | 0.0064 | 0.0046 | 0.0106 | 0.0029 | 0.0132 | 0.0101 | - | 0.0172 | 0.0019 | 0.0186 |
|  | I | 0 | 0 | 0 | 0 | 0 | 1 | 0 | 3 | - | 2 | 2 | 1 |

L: length of fragment; π: nucleotide diversity per site; I: number of indels and inversions
