# Supplementary material for: Highly Variable Chloroplast Markers for Evaluating Plant Phylogeny at Low Taxonomic Levels and for DNA Barcoding
Source: PLoS One. 2012 Apr 12;7(4):e35071. doi: 10.1371/journal.pone.0035071 (PMC3325284; doi:10.1371/journal.pone.0035071)
Supplement: Table S2 — Samples used to test the of 23 chloroplast loci. (DOC) [file pone.0035071.s002.doc]

Table S2. Samples used to test the of 23 chloroplast loci.

| Taxon | Locality | Voucher | Remark |
| --- | --- | --- | --- |
| *Chimonanthus praecox* (L.) Link | Beijing Botanical Garden, CAS, Beijing, China | S.L. Zhou 0020 | AS test |
| *Typha orientalis* Presl. | Beijing Botanical Garden, CAS, Beijing, China | J. H. Xue 090923A | AS test |
| *Paeonia suffruticosa* Andrews | Luoyang, Henan, China | BOP | AS test |
| *Panax bipinnatifidus* Seem. | Ninglang,Yunnan,China | WY2023502 | AS, SS and V tests |
| *Nelumbo nucifera* Gaertn. | Beijing Botanical Garden, CAS, Beijing, China | J. H. Xue | AS, SS and V tests |
| *Nelumbo lutea* Willd. | Columbus County, NC, USA | BOP | AS, SS and V tests |
| *Prunus persica* (L.) Batsch | Zhengzhou, China. | QX048 | AS, SS and V tests |
| *Prunus mira* Hoehne | Chayu, Tibet, China | QX138 | AS, SS and V tests |

AS: amplification success; SS: sequencing success; V: variability.
