# Supplementary figures and images for: Highly Variable Chloroplast Markers for Evaluating Plant Phylogeny at Low Taxonomic Levels and for DNA Barcoding
Source: PLoS One. 2012 Apr 12;7(4):e35071. doi: 10.1371/journal.pone.0035071 (PMC3325284; doi:10.1371/journal.pone.0035071)

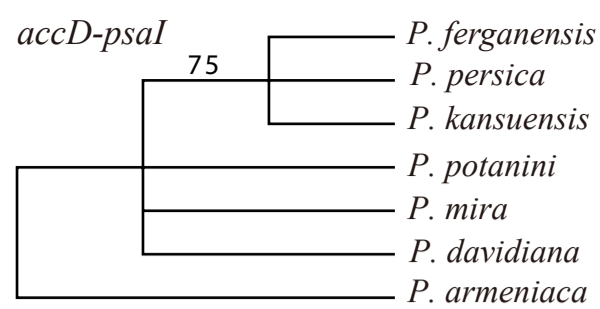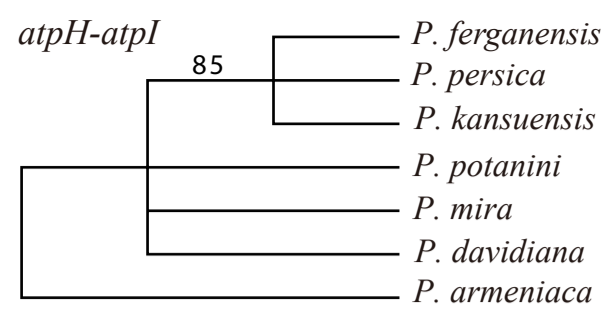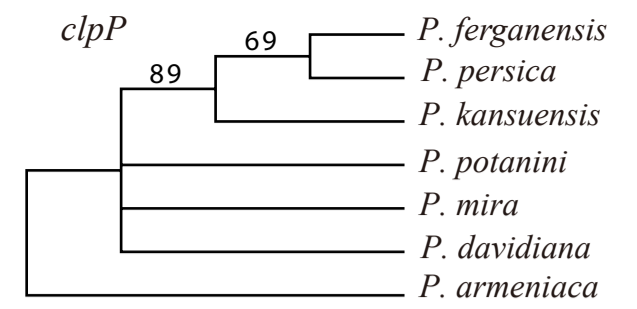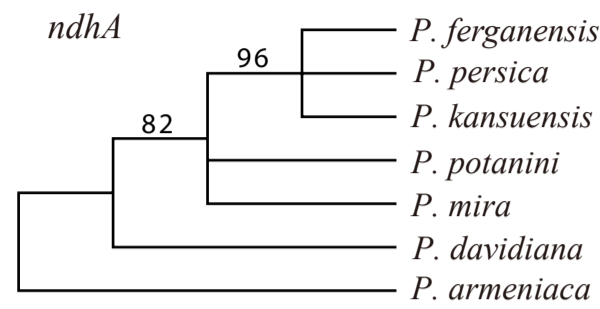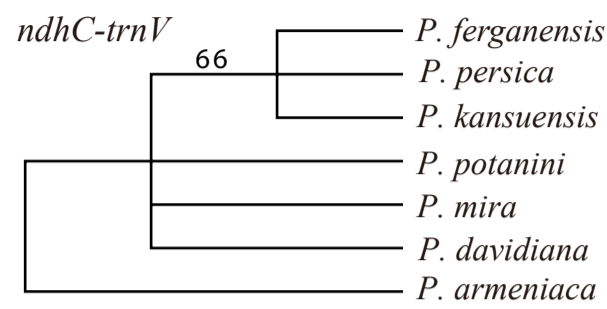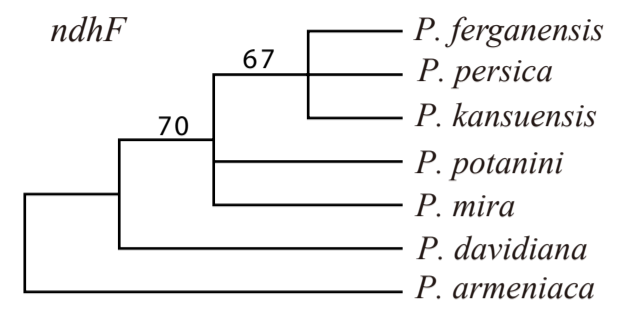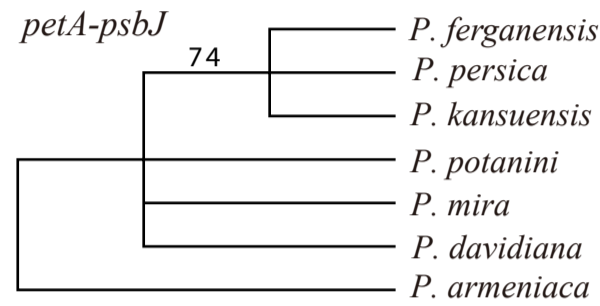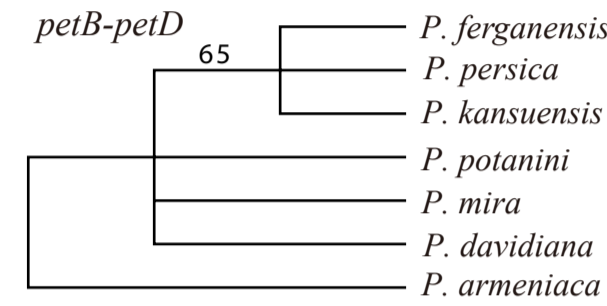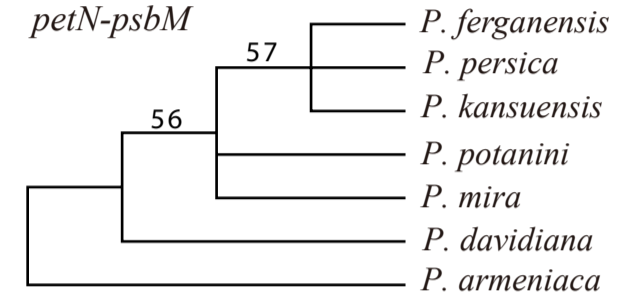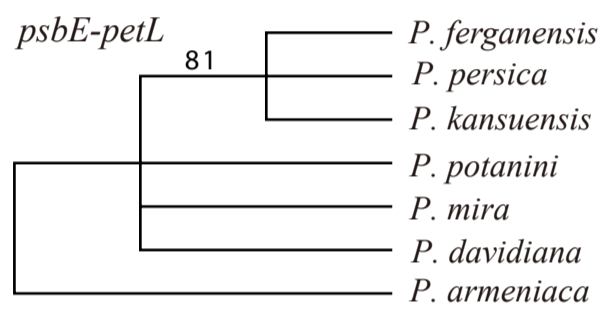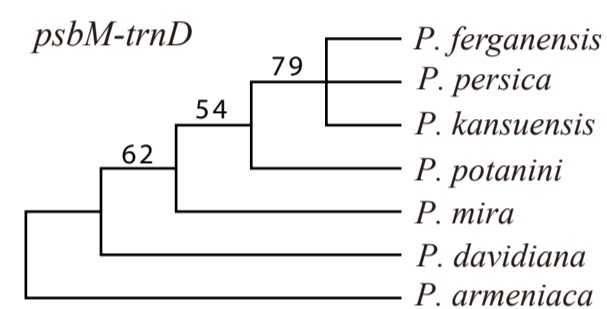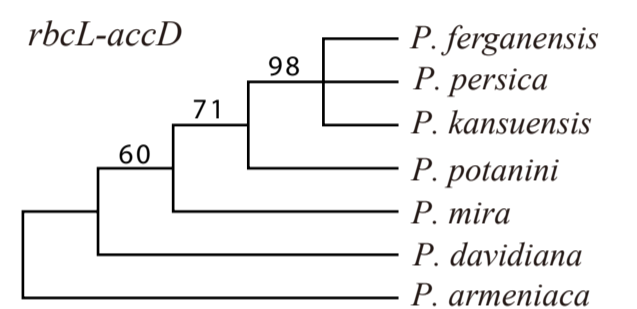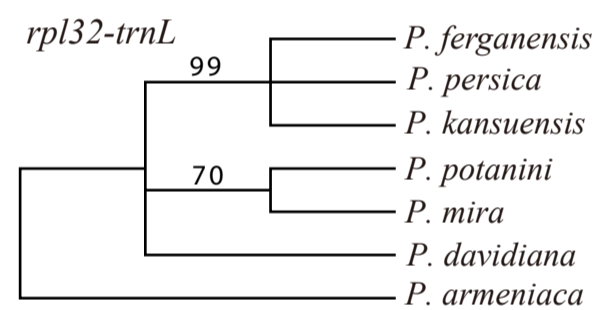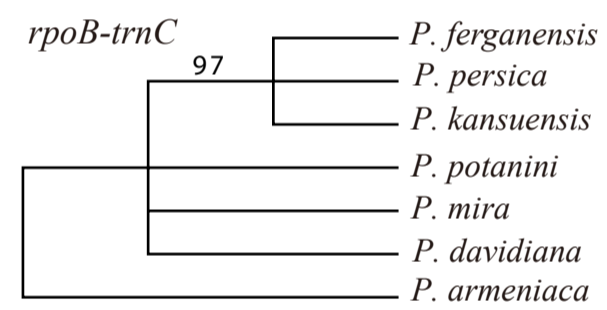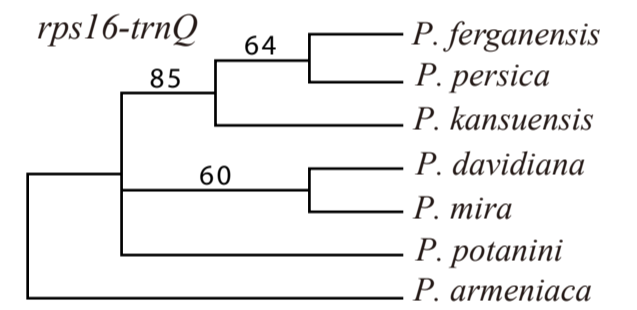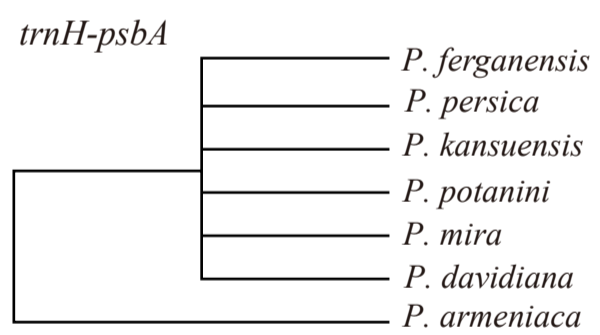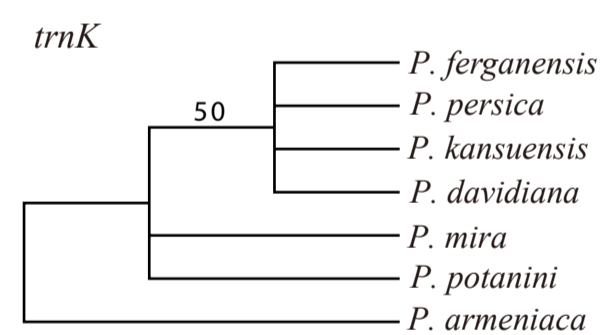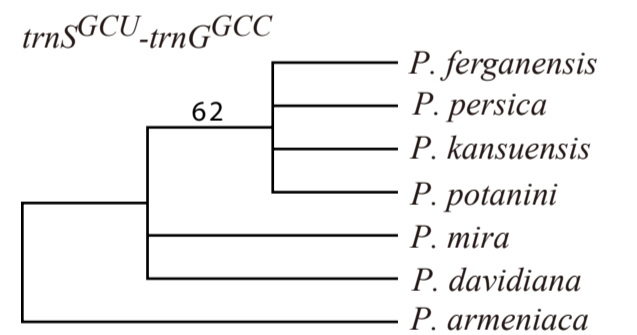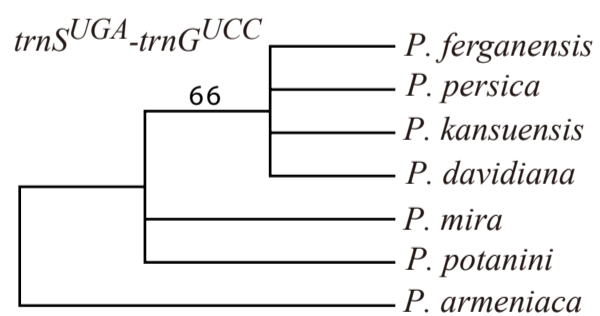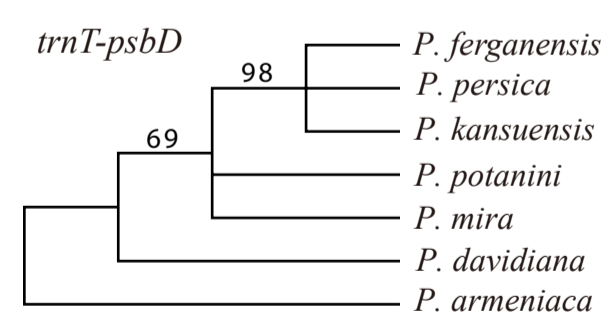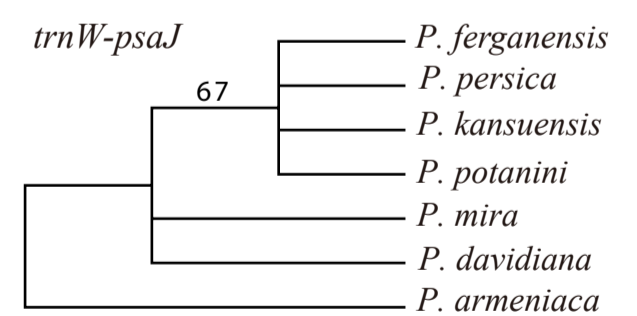

Supplement: Figure S1 — Maximum parsimony trees of all six peach species (Prunus sect. Persica) based on 21 chloroplast loci, showing the resolutions of the loci in the group. The figures above the lines are the bootstrap values for the clades. (PDF) [file pone.0035071.s004.pdf]
